# Gut immunity in a protochordate involves a secreted immunoglobulin-type mediator binding host chitin and bacteria

**DOI:** 10.1038/ncomms10617

**Published:** 2016-02-15

**Authors:** Larry J. Dishaw, Brittany Leigh, John P. Cannon, Assunta Liberti, M. Gail Mueller, Diana P. Skapura, Charlotte R. Karrer, Maria R. Pinto, Rosaria De Santis, Gary W. Litman

**Affiliations:** 1 Department of Pediatrics, University of South Florida Morsani College of Medicine, Children's Research Institute, 140 7th Avenue South, St Petersburg, Florida 33701, USA; 2 College of Marine Sciences, University of South Florida St Petersburg, 140 7th Avenue South, St Petersburg, Florida 33701, USA; 3 Department of Biology and Evolution of Marine Organisms, Stazione Zoologica Anton Dohrn, Villa Comunale, Napoli 80121, Italy; 4 Department of Molecular Genetics, All Children's Hospital Johns Hopkins Medicine, 501 6th Avenue South, St Petersburg, Florida 33701, USA

## Abstract

Protochordate variable region-containing chitin-binding proteins (VCBPs) consist of immunoglobulin-type V domains and a chitin-binding domain (CBD). VCBP V domains facilitate phagocytosis of bacteria by granulocytic amoebocytes; the function of the CBD is not understood. Here we show that the gut mucosa of *Ciona intestinalis* contains an extensive matrix of chitin fibrils to which VCBPs bind early in gut development, before feeding. Later in development, VCBPs and bacteria colocalize to chitin-rich mucus along the intestinal wall. VCBP-C influences biofilm formation *in vitro* and, collectively, the findings of this study suggest that VCBP-C may influence the overall settlement and colonization of bacteria in the *Ciona* gut. Basic relationships between soluble immunoglobulin-type molecules, endogenous chitin and bacteria arose early in chordate evolution and are integral to the overall function of the gut barrier.

## Abstract (web summary)

Protochordates, including *Ciona intestinalis*, lack an adaptive immune system but possess innate immune receptors, including the secreted immunoglobulin V-region-containing VCBPs. Here the authors show that VCBP-C of *Ciona* binds gut bacteria and chitin-rich gut mucosa, influences biofilm formation and likely plays a role in gut homeostasis.

## Graphical abstract

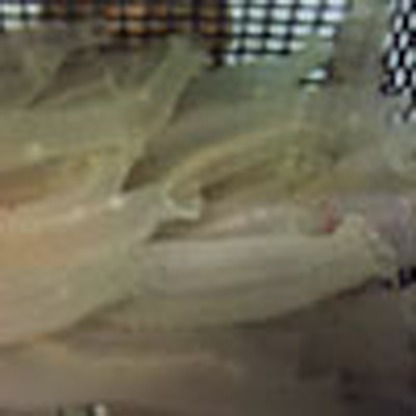

Protochordate species lack an adaptive immune system but possess multigene families of innate immune receptors^1^, including the secreted immunoglobulin variable region-containing chitin-binding proteins (VCBPs)^2^, which are not found in vertebrates. Unlike V-region-containing antibodies and T-cell antigen receptors, the VCBPs do not undergo somatic rearrangement but some exhibit regionalized hyperpolymorphism^3^. The V-region domains^4^ of VCBPs bind and promote the opsonization of bacteria^5^; the distinctive C-terminal chitin-binding domain (CBD) likely is also integral to overall function^2^,^5^,^6^.

The expression of VCBP genes in both *Branchiostoma floridae* (amphioxus)^2^ and *Ciona intestinalis* is confined largely to the gut^5^,^6^, where distinctive patterns of both spatial and temporal expression of VCBPs during development are seen^6^. Genes encoding VCBPs in *Ciona* are expressed abundantly at early stages in the juvenile, corresponding to the development of both the stomach and intestinal compartments and preceding the onset of feeding^6^; VCBPs represent an early marker of gut development. VCBPs are expressed primarily in the gut epithelium and are secreted into the lumen where they bind bacteria^5^.

Here we show that endogenous expression of chitin occurs within the gut of *Ciona* and that this chitin is an integral component of gut-specific mucus. The expression of VCBP-C can be detected from the earliest stages of development; VCBP-C and ultimately bacteria both bind and colocalize to the resulting chitin-rich mucus matrix. VCBPs, through association with an extensive network of chitin fibrils, may influence settlement of bacterial communities by modulating adherent biofilms on epithelial surfaces. Thus, in chordate taxa that diverged before the evolutionary emergence of adaptive immunity, soluble immune mediators encoding V-type immunoglobulin domains likely serve a role in the establishment and maintenance of gut homeostasis by modulating bacterial community structure.

## Results

### The epithelium-associated mucus is chitin-rich

**Figure 1:**
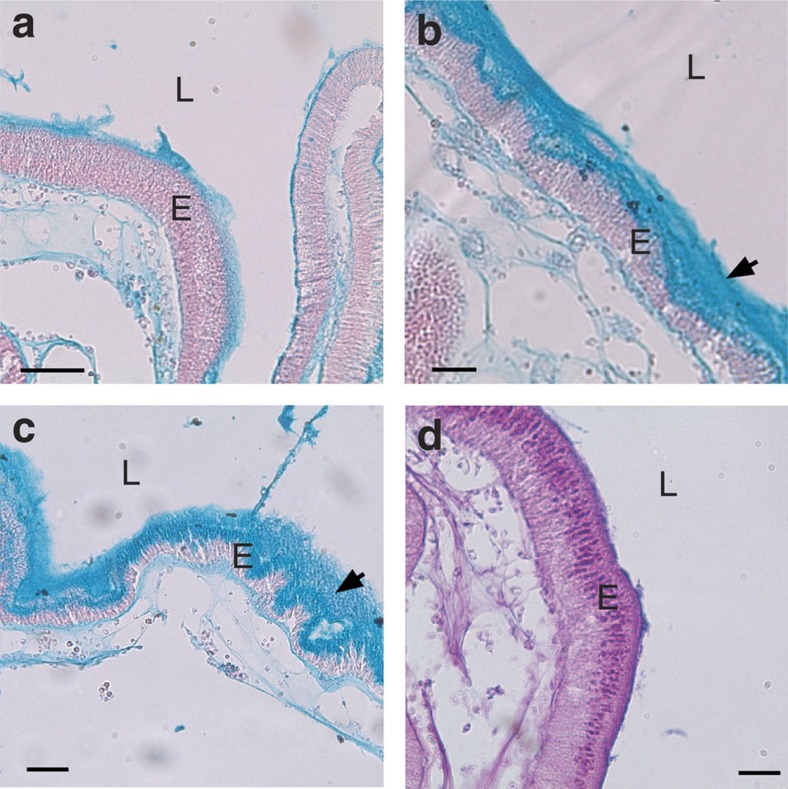
Two types of epithelium-associated mucus line the *Ciona* gut. A thin layer of mucus (**a**) covers the epithelium of the stomach in the adult gut, while thicker mucus (**b**,**c**) is found in the mid- to distal-gut epithelium. (**a**–**c**) Alcian blue staining suggests abundant acid mucopolysaccharides. (**d**) Staining with Alcian blue/periodic acid-Schiff identified neutral polysaccharides that were confined mostly to the intracellular vacuoles of the secretory epithelial cells forming the gut walls. Sections (**a**–**c**) were counterstained with nuclear fast red. Scale bars (**a**,**c**), 50 μm and (**b**,**d**) 25 μm. Arrows indicate two types of mucus: dense and layered (**b**) and loosely associated (**c**). E, epithelium; L, lumen.

Staining of paraffin-embedded intestinal sections with Alcian blue indicates that the mucus lining the gut epithelium of adult *Ciona* consists primarily of acidic mucopolysaccharides (Fig. 1a–c)^7^, a characteristic of vertebrates^8^. The layer immediately adjacent to the epithelium is rigid and resembles the intestinal glycocalyx of mammals, whereas that facing the lumen consists either of a densely woven layer of mucus, which is thinner in the stomach (Fig. 1a and Supplementary Fig. 1a) and thicker throughout most of the midgut (Fig. 1b) or loose/more dispersed in the remaining distal gut areas (Fig. 1c). The latter form is prone to becoming dislodged during histological processing, and its appearance also may result from offset or angled sections (incorporating intestinal curvature). Both forms of mucus have been described in mammals^9^. Thick ribbon-like chitin-rich mucus often is seen accumulating at the base of the stomach epithelial folds (Supplementary Fig. 1b), consistent with the stomach epithelial expression patterns of VCBP-C (Supplementary Fig. 1c)^6^. Staining with Alcian blue/periodic acid-Schiff identified neutral polysaccharides that were confined mostly to the intracellular vacuoles of the secretory epithelial cells forming the gut wall (Fig. 1d).

**Figure 2:**
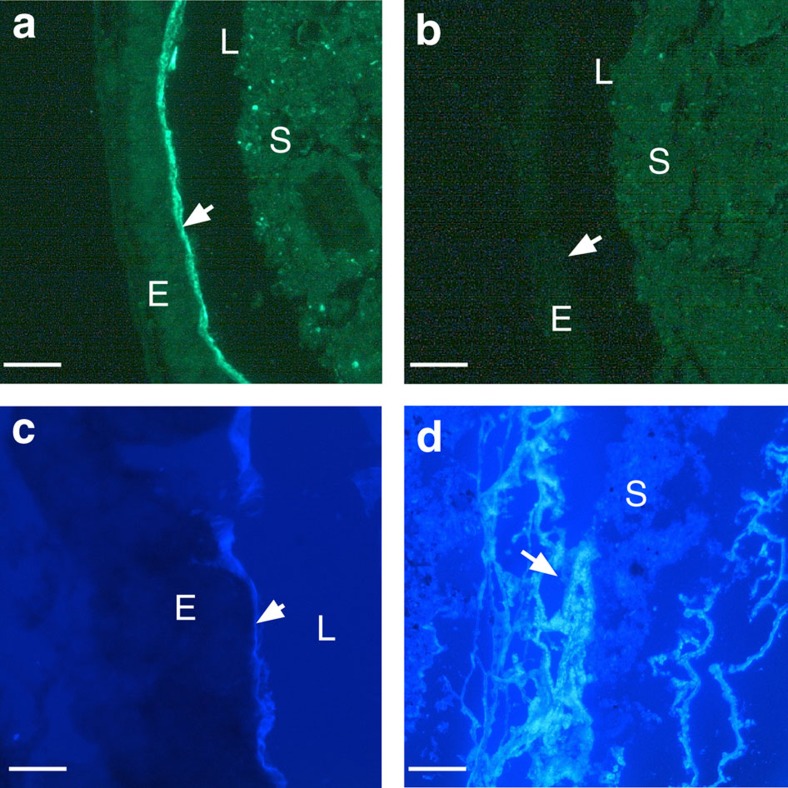
Detection of chitin within *Ciona* gut mucus. (**a**) Chitin was detected initially (arrow) within the mucus throughout the gut by staining with Fc-CBD-C DyLight 488 (green) and (**b**) subsequently was depleted by treatment with chitinase. The absence of Fc-CBD signal also is achieved by pre-treating tissue sections with chitinase. (**c**) Calcofluor white staining confirmed the presence of chitin at the epithelial surface (arrow); copious amounts of loose chitin-rich mucus often are detectable in the lumen (**d**). Identical chitin staining patterns were detected with Alexa Fluor 488-coupled chitin-binding protein (New England BioLabs; Supplementary Fig. 1f). Staining with isotype and secondary antibody controls was negative. Scale bars (**a**–**c**), 25 μm and (**d**) 100 μm). E, epithelium; L, lumen; S, stool.

A recombinant Fc-chimeric probe derived from the CBD of VCBP-C (Fc-CBD-C) revealed prominent immunohistochemical (IHC) staining of epithelium-associated mucus lining the gut wall (Fig. 2a and Supplementary Fig. 1a), extending from the stomach through the midgut and hindgut. This signal can be diminished with chitinase treatment (Fig. 2b). Notably, the abundant mucus that is associated with the branchial basket largely is to staining with Fc-CBD-C, suggesting that most chitin production begins downstream of the pharynx. Indistinguishable staining patterns were observed using calcofluor white (Fig. 2c,d and Supplementary Fig. 1d), a chitin-specific general histological stain, as well as a different recombinant chitin-binding protein probe (Alexa Fluor 488-CBP, New England Biolabs; Supplementary Fig. 1e,f). Copious amounts of free, chitin-rich, mucus often are seen in the gut lumen (Fig. 2d); a chitin-rich glycocalyx encasing fecal pellets throughout the intestinal compartments also is evident (Supplementary Fig. 2).

### VCBP-C and bacteria colocalize at surface of the gut epithelium

**Figure 3:**
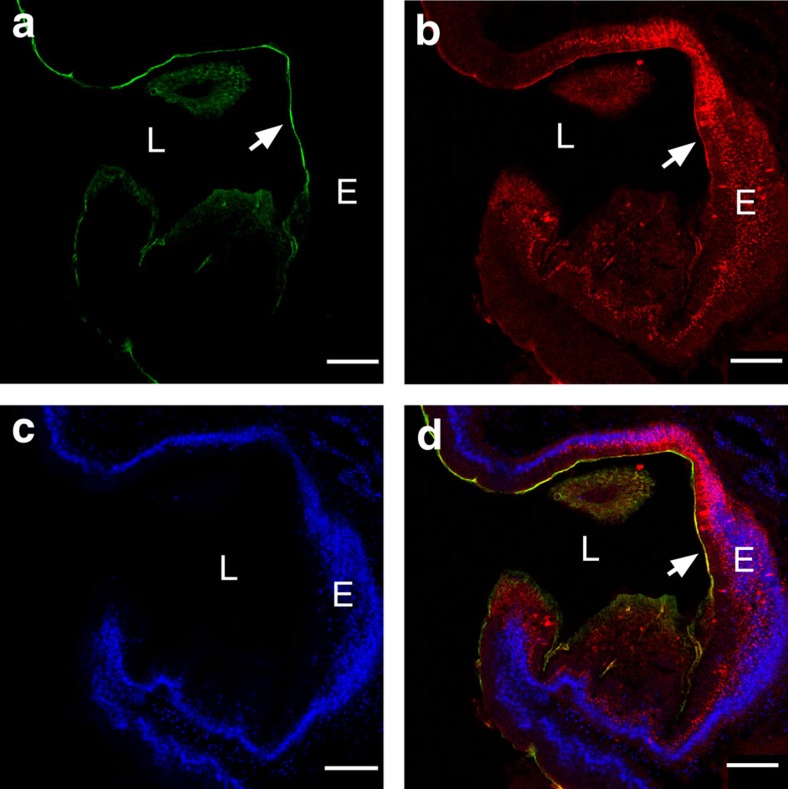
VCBP-C colocalizes with chitin-rich mucus at the surface of the stomach epithelium near the midgut and is visualized with confocal microscopy. (**a**) Chitin staining (arrow) detected by Fc-CBD-C DyLight 488 (green), (**b**) VCBP-C staining (arrow) detected by Alexa Fluor 594 (red) at the surface mucus as well as within granules of the epithelium, (**c**) Hoechst staining of DNA and (**d**) merged (arrow; overlay indicated in yellow). Staining with isotype and secondary antibody controls at varying concentrations was negative. Scale bars, 50 μm. E, epithelium; L, lumen.

**Figure 4:**
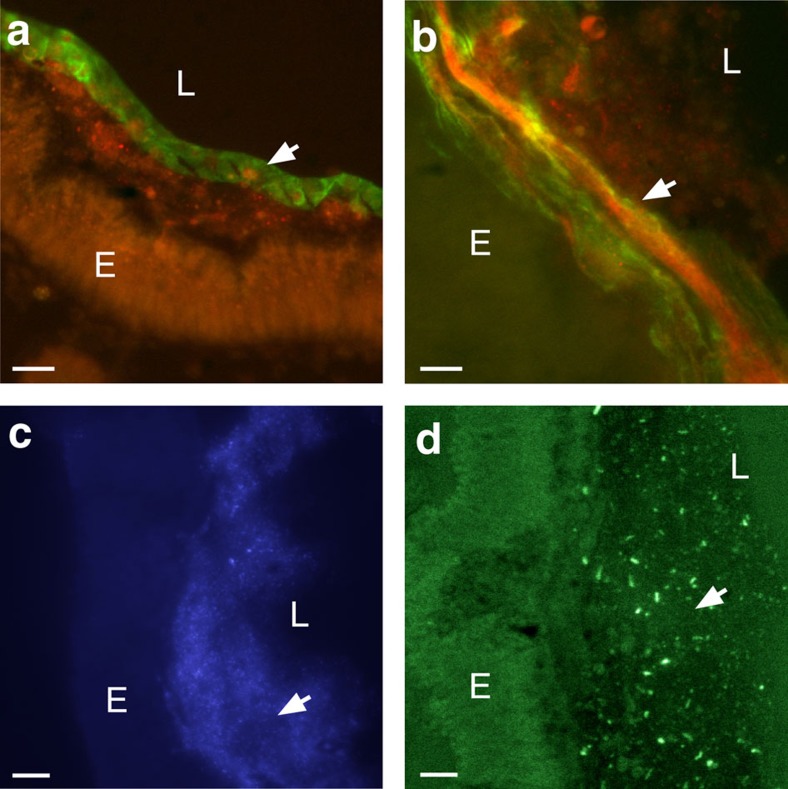
Detection of VCBP-C in both dense and loosely associated mucus. (**a**,**b**) Colocalization (yellow-merged signal) of chitin (Fc-CBD-C DyLight 488, green) and VCBP-C (Alexa Fluor 594, red). Dense mucus (glycocalyx-like) of the midgut can form (**a**) ribbon-like structures (arrow) as opposed to (**b**) less dense mucus (arrow) seen in the distal gut. (**c**) Microbiota-sized particles (arrow) seen in the mucus detected by Hoechst staining of DNA were confirmed as bacteria by 16S FISH (arrow) (**d**). Staining is negative with isotype and secondary antibody controls. Scale bars,10 μm. E, epithelium; L, lumen.

Double immunofluorescent staining of gut mucus with the Fc-CBD reagent as well as with antibody directed to the V2 domain of VCBP-C is illustrated in Figs 3 and 4. VCBP-C has been shown previously to be expressed by the gut epithelium, stored in granules and released into the lumen^5^. VCBP-C and chitin-rich mucus are shown here to colocalize to the surface of the gut epithelium (Fig. 3). VCBP-C colocalizes to chitin fibrils within mucus but also can be detected in other chitin-deficient, mucus-rich areas of the gut, as well as under the glycocalyx, in more intimate contact with the epithelium. This latter effect is most apparent where the glycocalyx has become partially detached during tissue-processing (Fig. 4a and Supplementary Fig. 3a,b). VCBP-C colocalizes to mucus even in the adult midgut, in which the epithelium is not known to secrete significant amounts of VCBP-C; the interaction of VCBP-C with the chitin matrix may originate in upstream gut compartments. The sparse or loose mucus found in the distal gut (Fig. 1c) often is interwoven with loosely packed chitin fibrils (Fig. 4b) and, correspondingly, the reactivity with antibody to VCBP-C can appear interspersed. VCBP-C also is found in the gut lumen where it previously was shown to interact with dietary contents that include bacteria^5^. It is likely that in adult animals a portion of the secreted VCBP-C is captured at the surface mucus (Fig. 4a,b and Supplementary Fig. 3c) through interaction with chitin, and another portion enters the lumen.

Bacteria, which associate with surface mucus and can be visualized by DNA staining using acridine orange or Hoechst dyes (Fig. 4c and Supplementary Fig. 3d), colocalize with the VCBP-C signal in the mucus layers (Fig. 4d and Supplementary Fig. 3d–f). Microbial cells appear in either sparse or dense clusters throughout the gut mucus but are most abundant in the loosely packaged mucus of the distal gut (Fig. 4c and Supplementary Fig. 3d). The presence of microbes along the gut wall varies by compartment and is not necessarily continuous. The DNA stains also produce microbe-free signals in some mucosal areas, which likely represent reactivity with extracellular DNA integrated into biofilms^10^. Most of the bacterial signals are consistent with the fluorescent *in situ* hybridization patterns produced by a probe complementing 16S genes (Eubacterial 16S probe cy3-338; Fig. 4d)^11^. Sparse organization of distal mucus is consistent with observations in mammals, in which bacterial and host-associated metabolic activity often contribute to loosened mucus^12^.

### VCBP-C and chitin colocalize in early non-feeding gut

**Figure 5:**
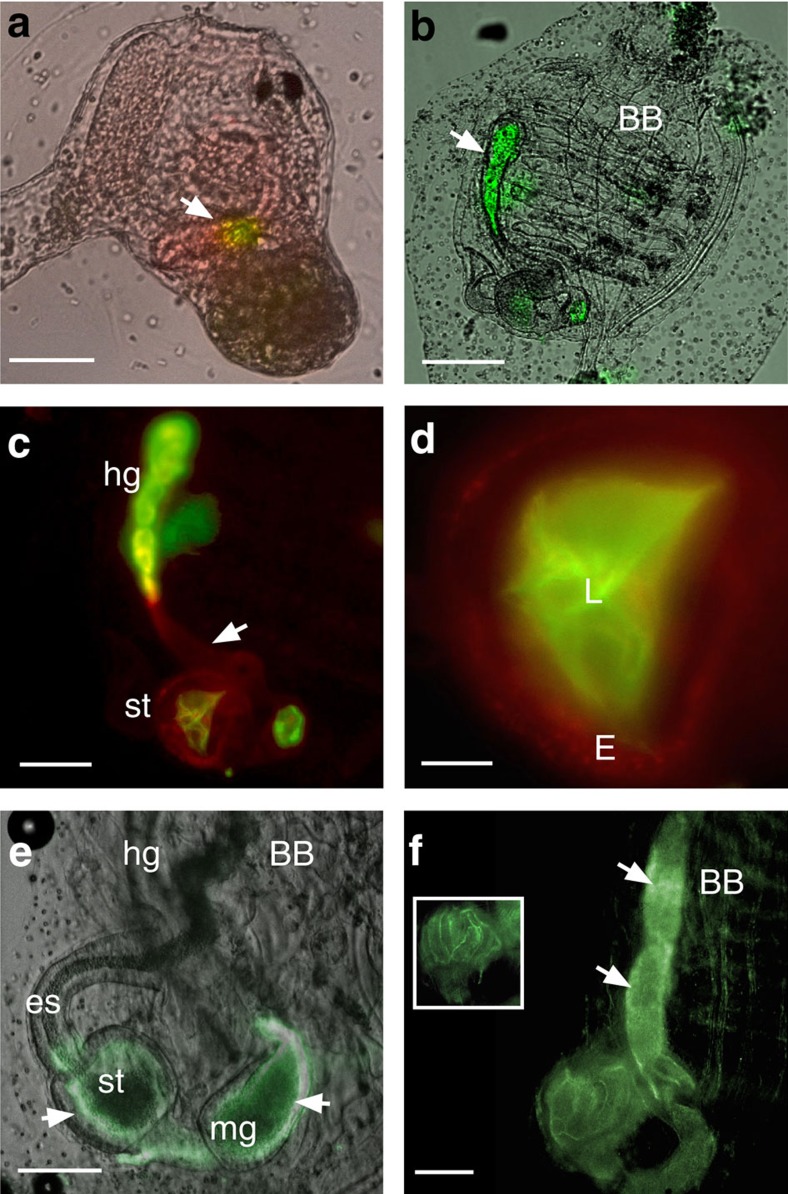
Chitin is expressed endogenously and its colocalization with VCBP-C is independent of microbial exposure. (**a**) Signals for VCBP-C (Alexa Fluor 594, red) and chitin (Fc-CBD-C DyLight 488, green) are colocalized (yellow, arrow) in the intestinal region of the gut primordium of late rotation stage juveniles maintained under germ-free conditions and persists throughout development. Gut development is complete by stage 5/7, with the immediate onset of feeding^13^. (**b**) Intestinal mucus is chitin-rich (in green, arrow). (**c**) Magnified view of gut (from **b**) in which VCBP-C (in red, arrow) is distributed primarily at the edges of the gut tissue surfaces. Chitin-rich pellets are seen in the distal gut and are purged into environment before feeding. (**d**) Magnified view of the stomach demonstrates chitin-rich mucus and VCBP-C in the epithelium, red. (**e**) Chitin (green) is prominent and restricted to the stomach and midgut epithelium 2–3 weeks post fertilization; chitin-rich mucus cannot be detected in the oesophagus or branchial basket. Intense signal is evident at the outer edges and are more prominent on the ventral side (arrows) of the stomach and midgut; a chitin signal also is prominent in fecal pellets (not visible in **e**) but cannot be detected in the hindgut epithelium. (**f**) Chitin is prominent throughout the gut to the anus in the whole-mount staining of young adults (second ascidian stage and onwards); images of both sides of the stomach are included to emphasize enhanced signal in ridges of the epithelial grooves (inset). Bright field overlays are shown in **a**,**b**,**e**. Scale bars (**a**,**c**), 50 μm; (**b**,**e**), 100 μm; (**d**) 25 μm; and (**f**) 200 μm. BB, branchial basket; E, epithelium; es, oesophagus; hg, hindgut; L, stomach lumen; mg, midgut; st, stomach.

*Ciona* represents a particularly well-defined model of chordate development^6^,^13^ (http://bioinfo.s.chiba-u.jp/ciaba/). Juveniles initiate feeding during late stages of metamorphic development. Different compartments of the digestive tract such as the oesophagus, stomach and intestines can be discerned histologically at stage 5 of the first ascidian stage juvenile. To determine whether the chitin found in the gut is of endogenous or exogenous origin, whole-mount immunofluorescent staining was used to localize chitin during various stages of metamorphosis before or after the initiation of feeding. At the late rotation stage, chitin and VCBP-C colocalize in the tube-shaped structure of the developing intestine (Fig. 5a) and fill the gut lumen later in development (Fig. 5b–e). At stage 7 of the first ascidian stage juvenile, chitin and VCBP-C are detected and mucus can be seen in the lumen (Fig. 5b–d); both signals colocalize in the stomach and intestine where chitin-rich mucus forms pellet-like structures even before the onset of feeding. A consistent pattern of VCBP-C expression throughout most of the gut is evident in whole-mount embryos from the late rotation stage onwards. VCBP-C and chitin staining are colocalized when mucus begins to fill the lumen. *Ciona* juveniles begin to acquire adult-like morphological features^14^ at stages corresponding to 12–20 days post settlement (last half of the first ascidian stage), during which final development of the branchial basket occurs and abundant expression of chitin in the stomach and midgut can be detected by staining with Fc-CBD-C (Fig. 5e). In these more mature juveniles, the stomach, a major site of mucus and chitin production, possesses a thick layer of chitin-rich mucus (and a small-volume lumen) where VCBP-C is found to colocalize strongly (Supplementary Fig. 4a–c). A prominent unilateral signal is evident on the ventral side of the gut wall (Fig. 5e and Supplementary Fig. 4a); in the distal gut, chitin staining is restricted to fecal pellets. Fecal pellets appear to become coated by a chitin-rich glycocalyx as the pellet exits the stomach (Supplementary Fig. 4d,e); long fibres of chitin-rich mucus are secreted in juveniles (Supplementary Fig. 4d,e) and adults (Supplementary Fig. 4f). After day 20 of development (second ascidian stage), chitin staining fills the entire gut from stomach through to the anus (Fig. 5f).

Expression of chitin synthase in the stomach and midgut areas can be detected by RNA *in situ* hybridization in early stages of development (Supplementary Fig. 5). Because chitin can be detected in juveniles before the onset of feeding, the expression and colocalization of both chitin and VCBP-C are not coupled to dietary sources and are independent of bacterial exposure.

### The presence of VCBP-C modulates biofilm formation *in vitro*

Most bacterial members of the gut microbiome colonize and reside as adherent communities by forming biofilms^15^,^16^, a process that is influenced by both biophysical properties and physiological conditions. Various host factors, which are secreted into the lumen by epithelial cells, can become trapped or immobilized in the mucus layers where they also can interact with colonizing flora and influence adherence and biofilm formation^17^,^18^,^19^. The presence of chitin at the mucosal surfaces of *Ciona* is unlikely to be bacteriolytic on its own; however, it may provide both a potential carbon source and a structural matrix for biofilm formation for a subset of gut microbes. The same chitin also may serve as a physical barrier to colonization by other microbes. Because VCBPs and chitin colocalize to the host epithelial-associated mucus so conspicuously, a further question arises as to whether or not the formation of the biofilms is influenced by VCBPs directly.

**Figure 6:**
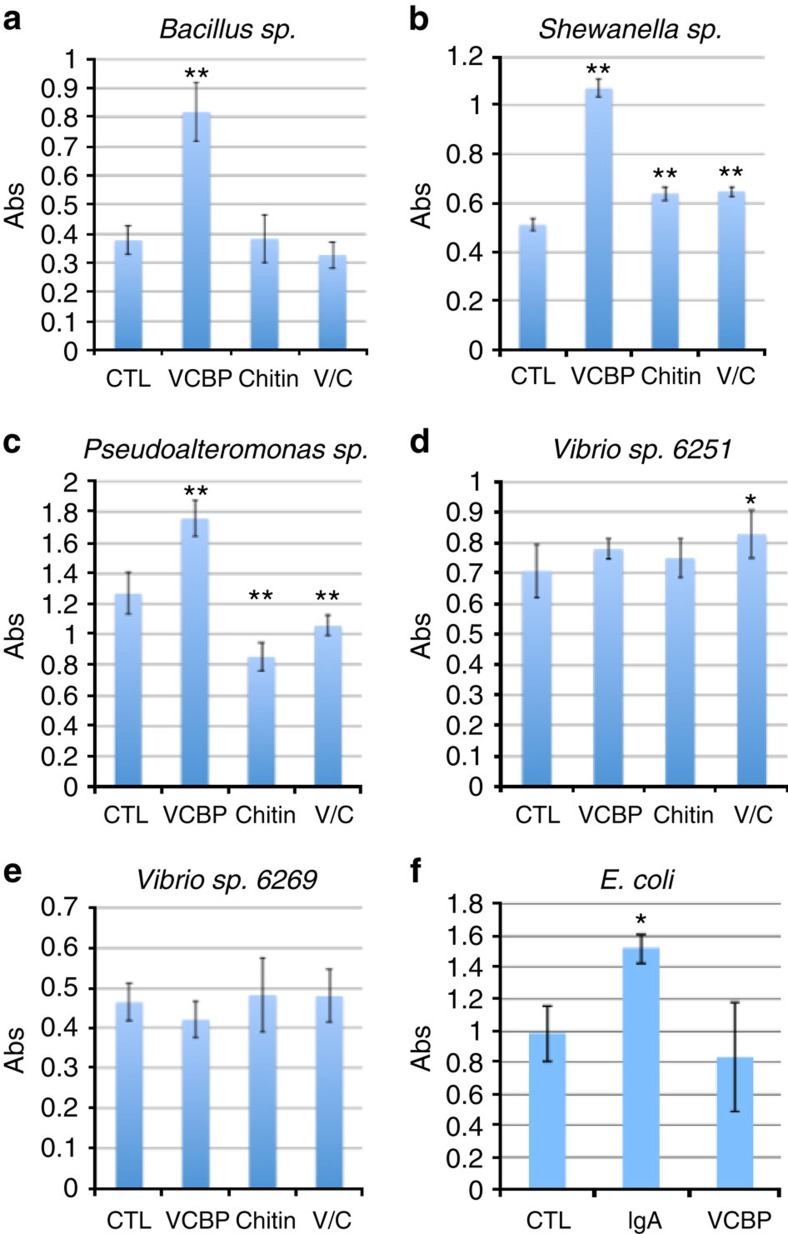
VCBP-C affects *in vitro* biofilm formation differentially in five gut bacterial isolates recovered from *C. intestinalis*. (**a**) Stationary cultures of a *Bacillus sp*. isolated from the *Ciona* gut forms biofilms within 3–5 days. Biofilm formation is enhanced in the presence of recombinant VCBP-C (6.5 μg ml^−1^); a similar effect is noted for gut isolates of (**b**) *Shewanella sp.* and (**c**) *Pseudoalteromonas sp.* (**d**) *Vibrio* isolate 6251 (laboratory-assigned number) exhibits a significant increase only with the addition of VCBP-C plus chitin; (**e**) no significant difference is seen with *Vibrio* isolate 6269. (**f**) *E. coli* biofilms are increased in the presence of SIgA; VCBP-C at similar concentrations does not influence *E. coli* biofilms. Biofilms were stained with crystal violet, dried, re-dissolved in acetic acid and absorbance (Abs) was read at OD_550_. Increased absorbance reflects the increased surface biofilm. Each experiment was performed in triplicate a minimum of four separate times; results shown represent one triplicate experiment. s.d. is shown by black error bars. Significant differences from control samples were calculated by using analysis of variance with *post hoc* Dunnett's test. *P<0.05; **P<0.01; CTL, control; VCBP, VCBP-C; V/C, VCBP-C plus hydrolysed chitin.

**Figure 7:**
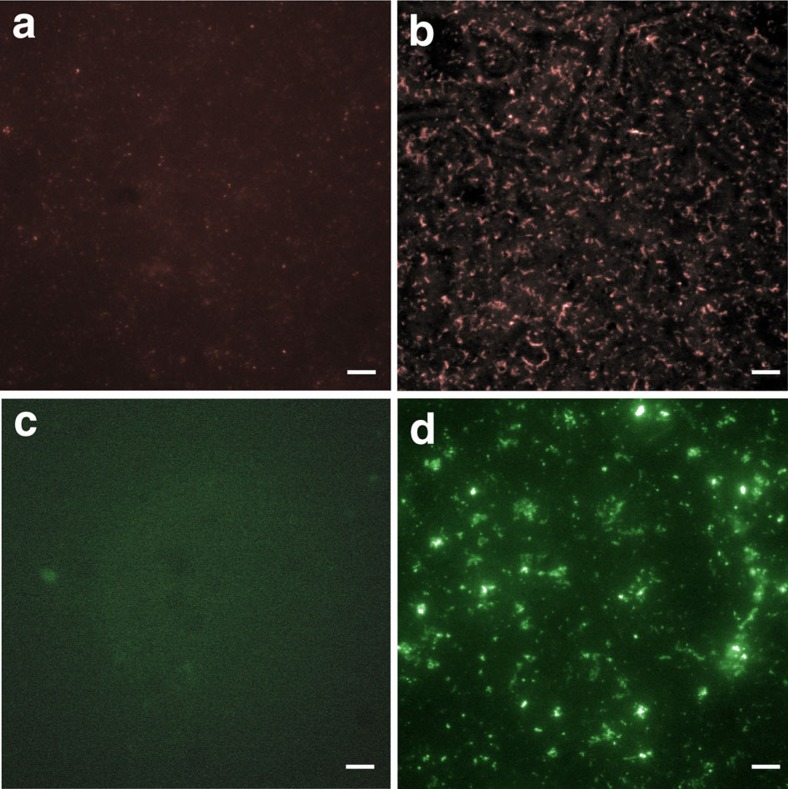
VCBP-C associates with bacteria in a *Shewanella sp.* biofilm. *Shewanella* were stationary-cultured in the presence of VCBP-C. Immunofluorescent staining detected VCBP-C bound to the bacteria of the biofilms using anti-VCBP-C (Alexa Fluor 594, red). (**a**) Control stain (no addition of VCBP-C) using primary and secondary antibodies, (**b**) VCBP-C-positive biofilm. SIgA influences biofilm formation in a non-pathogenic *E. coli* strain. (**c**) Control stain (no addition of SIgA) using fluorescently tagged anti-IgA antibodies and (**d**) SIgA-positive biofilm. Scale bars, 20 μm.

The potential role for VCBPs in modulating adherent biofilms was investigated by deriving single bacterial isolates from the gut of *Ciona*. Five bacterial species closely related to gut core operational taxonomic units^20^, including two *Vibrio sp.*, one *Pseudoalteromonas sp.*, one *Bacillus sp.* and one *Shewanella sp.*, were grown either in the presence or absence of hydrolysed chitin. Production of biofilms involves the formation of an exopolysaccharide-rich sheet that, *in vitro*, adheres to plastic dishes and can be stained and quantified with crystal violet^21^. Biofilm production was significantly increased in the *Bacillus sp*., *Pseudoalteromonas sp*. and *Shewanella sp*. with the addition of either affinity-purified^5^ or recombinant VCBP-C protein (6.5 μg ml^−1^; Fig. 6a–e). Hydrolysed chitin alone or in combination with VCBP-C had a significant influence on biofilm formation in *Shewanella sp.* (Fig. 6b) and *Pseudoalteromonas sp.* (Fig. 6c); however, the addition of hydrolysed chitin was not required for the observed effect of VCBP-C. Two of the *Vibrio* species tested exhibited no effect of VCBP-C on biofilm production; however, one species (6251; Fig. 6d) demonstrated a mild but significant enhancement in biofilms when exposed to both VCBP-C and hydrolysed chitin. The observed effects also have been confirmed with native, affinity-purified, VCBP-C. Stereomicroscopy using the ALI Assay (Supplementary Fig. 6)^22^ indicates that the increase in crystal violet staining (Fig. 6) corresponds with a general increase in surface coverage by the maturing, adherent, biofilm. The estimated concentration of VCBP-C from affinity isolation experiments^5^ in the stomach extract of adult *Ciona* is 15 mg ml^−1^. Enhancement of biofilm production by soluble VCBPs was concentration-dependent, and the effect was reversed by heat inactivation. VCBPs at concentrations ranging from 6.5 μg ml^−1^ to 0.5 mg ml^−1^ (the highest tested) influence biofilm formation by direct contact with bacteria within the biofilm (Fig. 7a,b). A recombinant VCBP-C lacking the CBD (V1V2) was tested at similar concentrations and found to be consistently less effective than full-length VCBP-C at inducing biofilms, suggesting a role for the CBD in biofilm modulation. The reduced effect of the V1V2 protein is demonstrated in both the ALI assay (Supplementary Fig. 6d) and by immunostaining (Supplementary Fig. 6e,f).

Previously, it was demonstrated that one of the many functions of secretory IgA (SIgA) in mammals involves the modulation of biofilm production^23^; histologically, SIgA can be found integrated into the mucus layers of the gut^18^,^24^,^25^. Non-pathogenic *Escherichia coli* have been induced to produce more abundant *in vitro* biofilms in the presence of SIgA^23^, suggesting that these biofilms could enhance barriers in the gut^24^,^25^,^26^. We reproduced the original findings, here quantified using the crystal violet staining approach (see Fig. 6f and Supplementary Fig. 7)^21^, and demonstrate that SIgA also is bound to the bacteria of the biofilm (Fig. 7c,d).

Extensive differences in the effects of both chitin and VCBP-C on biofilm formation and stability by different species of bacteria are consistent with their involvement in a selective process, especially considering that VCBP-C (as demonstrated for SIgA and *E. coli*) can bind directly to bacteria within an adherent biofilm (Fig. 7). These observations warrant future studies aimed at determining the role(s) of VCBP-C in modulating adherent properties and biofilm formation among distinct communities of bacteria that colonize the gut.

## Discussion

The gut of both vertebrates and invertebrates has evolved to house rich communities of microorganisms^16^. The host utilizes diverse mechanisms, including immune mediators, to protect it from invading pathogens while ignoring, not responding to or protecting certain beneficial microbes. The epithelium-associated mucus is an important component of gut barriers and abundant non-pathogenic bacterial communities colonize these surfaces^12^,^18^,^24^. Details remain lacking on the mechanisms by which both vertebrates or invertebrates modulate the composition of bacteria in mucosal barriers. In vertebrates, SIgA plays important roles^27^.

*Ciona* represents a potentially informative model system for understanding gut immunity^16^,^28^. Specifically, *Ciona* ingests food through a siphon system that forces relatively large volumes of water through an extensive branchial basket, in which gas exchange occurs and food particles are selected and sieved before entering the oesophagus and ultimately passing to the stomach and intestines. *Ciona* feeds on a diverse diet of fine and microscopic carbon sources (for example, phytoplankton and bacteria), but the gut is colonized by a distinct and presumably functionally relevant microbiome^20^,^28^. Additional advantages include the following: a relatively large size; striking histological resemblance of its gut to that of more advanced chordates; an experimentally tractable model system with a sequenced, streamlined genome; a well-defined developmental programme; ability to be reared under germ-free conditions until the second ascidian stage is reached; and long-established mariculture approaches^29^,^30^. *Ciona* VCBP-C interacts with bacteria, is opsonic for granular (gut) amoebocytes^5^ and exhibits patterned, compartmentalized expression that is established at the earliest stages of development^6^. The V domains bind bacteria and elicit opsonization; however, until now the role of the CBD in VCBP function is unknown.

Many species of arthropods^31^ as well as many vertebrates^32^, with the exception of mammals, produce endogenous chitin in the gut. The midgut surfaces in some protostome invertebrates (for example, *Drosophila*) are lined by peritrophic matrices^33^, which are rich in both chitin and peritrophins^34^, glycoproteins that possess CBDs. A dense gel-like layer termed the peritrophic matrix is formed^33^, which establishes a barrier along the gut epithelium^35^. The resulting surface is fortified against parasites and the peritrophic matrix also functions in subcompartmentalization (for example, when fragments encase stool pellets) of metabolic processing of dietary material. Chitin production may not be limited to the gut, as blood cells from various invertebrates have been implicated^36^. The occurrence of chitin in the gut of *Ciona* and other tunicates has been reported previously^37^,^38^; our data suggest further that in *Ciona* chitin-rich mucosal surfaces not only provide physically enhanced barriers but also may provide a surface where proteins with CBD domains can anchor. The recent description of chitin in the gut of non-mammalian vertebrates is of potential significance to host-barrier defense; however, no vertebrate immune mediators have yet been shown to associate with a host-associated chitin matrix, which may serve as a primary interface with the adherent microbiome. Here we show that since the earliest developmental stages of gut formation, colocalization of VCBPs with endogenous chitin occurs. Soon thereafter, chitin forms long parallel fibrils that are integrated with epithelial-associated mucins of the *Ciona* gut. VCBP-C colocalizes to this matrix (via the CBD domain) and, taking into account early observations on the phagocytic function of VCBP-C, it is reasonable to assume that the interaction of bacteria with this matrix is mediated via the V domains of VCBP-C. Additional roles for VCBP-C in host immunity and barrier defenses are suggested by the results of *in vitro* biofilm assays in which we show that VCBP-C protein also can affect the formation of biofilms. Thus, while hydrolysed chitin was demonstrated to have a minimal influence on biofilm formation (Fig. 6), the presence of the CBD of VCBP-C appears to serve an important role.

The mammalian gut is protected not only by physical barriers and other innate mucosal defenses but also by SIgA, which effectively can agglutinate and/or opsonize bacteria^27^. SIgA can influence the development and composition of the gut microbiome^39^,^40^,^41^. In mammals, SIgA has been shown to associate with mucosal barriers and also influence bacterial biofilm formation via mechanisms that remain to be determined^12^,^18^,^23^,^24^. Interestingly, the massive glycan complex that forms the core of the secretory component of SIgA is chitobiose-rich and interacts directly with bacteria^42^; however, the overall significance of this association to host defense is not known. Analogies can be drawn, albeit speculative, between the observed associations of VCBPs in the gut and SIgA of mammals. Specifically, the interaction of SIgA with bacteria in the lumen regulates adherence to the epithelial surface through immune exclusion, an integral component of barrier defenses in mucosal tissues^43^. Subsequently, based on *in vitro* experimental observations, it was proposed that non-pathogenic bacteria residing as adherent biofilm communities among mucin-rich epithelial surfaces were the result of SIgA colocalization and agglutination of some bacterial strains^18^,^26^. This observation was reproduced in our *in vitro* assays; as a control, we demonstrate that even at 2 mg ml^−1^ (the concentration at which SIgA is most active on *E. coli* biofilms *in vitro*), VCBP-C does not influence *E. coli* biofilm assembly, further demonstrating an unexpected discriminatory potential of the interactions shown (Supplementary Fig. 7). The observations described herein suggest that VCBPs in the lumen, as well as VCBP colocalized to epithelial surfaces, modulate adherence and biofilm formation at the surface of intestinal epithelium. These functional properties could be predicted to serve important roles in maintaining homeostasis and place VCBP-C at the host–environment interface where the settlement of some transient bacteria are regulated in a process that resembles immune exclusion by SIgA; modulating the formation of biofilms is likely essential for barrier stability^44^.

It remains unclear why some immune mediators, such as VCBP-C in protochordates or SIgA in mammals, would induce the formation of biofilms in some bacteria. It is possible that some bacteria common to the lumen induce biofilms in direct response to being bound by these immune mediators, which often induce agglutination or are effective as opsonins. With non-pathogens, one can also easily envision a scenario where this ‘arms race' eventually developed into mutualistic interactions where both the bacteria and the host (for example, by enhancing barriers) can benefit from the resulting biofilms.

Immunoglobulin-mediated adaptive immunity as we know it in vertebrate species has long been thought to have originated in the gut where it serves important roles in barrier functions and likely predated the emergence of the rearranging antigen-binding receptors^45^,^46^,^47^. Specific intermediates in the evolutionary pathways that ultimately gave rise to the adaptive receptors of jawed vertebrates may never be able to be traced definitively as such efforts are confounded by the rapid divergence and heterogeneity of these families of molecules as well as their relatively sparse sequence conservation. Immunoglobulin V-type domains are well known to function in the context of immunity; historically, CBDs most often have been associated with chitin utilization pathways. We herein provide evidence that CBDs can function as components of immune-type molecules that function at the interface of host and microbiome. It would appear that fundamental relationships between soluble V-region-containing molecules and the gut microbiome were established before the origins of vertebrates and the adaptive immune system.

## Methods

### Animals

Wild-harvested *C. intestinalis* were obtained from harbors in the San Diego vicinity (M-REP). Animals were shipped overnight, immediately acclimated into filtered seawater and, if necessary, placed under continuous light for harvesting of gametes for mariculture^5^,^6^,^20^.

### Histological preparations

Animal guts were cleared for 48–72 h in filtered seawater with water being changed every several hours, dissected and placed into 4% paraformaldehyde (in artificial seawater) overnight with gentle rocking at 4 °C. Tissues were prepared for either frozen or paraffin-sectioning. After fixation, tissues for frozen sectioning were washed 5 × in PBS, soaked for at least 2 h or overnight in 20% sucrose/PBS and subsequently embedded and frozen on dry ice in OCT freezing media (Fisher Scientific). Frozen blocks were sectioned at 8–20 μm in a cryostat. Fixed tissue was dehydrated through a gradient ethanol series and paraffin-embedded via standard methods without the use of vacuum. Paraffin blocks were sectioned at 6–12 μm. The mucus of the *Ciona* gut can resist some forms of traditional histological processing, likely due to the fixation of mucus that is interwoven with polymerized chitin fibrils and bacterial biofilms (see below). Individual-specific modifications in handling and washing were introduced in the procedure to optimize results, including suturing the anterior and posterior ends of the gut, fixation of the gut with minimal handling, lowering of dissected gut compartments into fixative with rocking gently overnight at 4 °C and adopting additional precautions when handling the subsequent sections and slides. When possible, smaller animals were fixed and sectioned as whole animals, that is, without dissection, resulting in minimal disruptions to the gut architecture.

### Mariculture and development of germ-free animals

Animals were cultured and maintained under germ-free conditions according to slightly modified methods originally established for zebrafish^48^. The outer tunic of *Ciona* was brushed with ethanol and povidone iodine. Eggs and sperm were isolated from ducts by surgical manipulation in a laminar flow hood. Following *in vitro* fertilization, embryos were handled in 100-μm mesh baskets. To circumvent handling complications with embryos that had shed the chorion after sterilization, culture dishes were coated with 1% agarose and treated with ultraviolet light for 1 h while drying. Germ-free embryos were then allowed to develop into swimming larvae before being transferred to uncoated Petri dishes that were maintained in bench top clean hoods. Animals were maintained in 50–100-IU ml^−1^ of penicillin and 50–100 μg ml^−1^ of streptomycin for at least the first week. Culture media and/or artificial seawater, as well as random animal samples, were checked weekly for contamination by monitoring growth on media that support the growth of a variety of marine microorganisms, including marine, tryptic soy, nutrient and brain–heart infusion agars, all with and without sea salts. Culture media also were screened with 16S PCR using 27F and 1492R primers^49^,^50^.

### Expression of recombinant VCBP-C forms

Construction of a chimeric human IgG1 Fc-CBD reagent was performed as previously described^51^ where cDNA sequences encoding the CBD (region 265–303 of NP_001190979) of VCBP-C were subcloned into the pcDNA3.0 vector. The human IgG1 Fc was positioned to be 5′ to the CBD sequence. Recombinant (Fc-CBD-C) protein was expressed in HEK293T cells (ATCC, CRL-3216); supernatants were harvested and Fc-CBD-C fusion protein was harvested using affinity purification with Protein A. Recombinant protein was washed and concentrated on Amicon spin columns (Millipore). The primary specificity of the reagent and reactivity with the second antibody were evaluated and confirmed.

Recombinant VCBP-C or V1V2 (CBD minus) proteins were expressed in bacteria, refolded and purified using previously established conditions^5^. Briefly, a cDNA fragment encoding the mature secreted form of VCBP-C was expressed in *E. coli* Tuner cells (Novagen) and inclusion bodies were isolated after lysis in detergent with added lysozyme and nuclease (Bugbuster and Lysonase; Novagen). Isolated inclusion bodies were denatured in 8-M guanidine, reduced using immobilized TCEP (Pierce Biotechnology) and refolded in 1 M guanidine/0.88 M arginine/2 mM glutathione. After dialysis against 50 mM NaCl/10 mM Tris pH 8.0, refolded VCBP-C was purified with fast protein liquid chromatography using a HiLoad 16/60 Superdex 75 gel filtration column (GE Healthcare Life Sciences). Purified protein was verified with SDS–PAGE and stored in aliquots at −80 °C in 50 mM NaCl/10 mM Tris pH 8.0.

### Histological and immunohistochemical staining

Established protocols were followed for histochemical staining of intestinal mucus with Alcian blue (0.5–2 h in 1 mg ml^−1^, pH 2.5 using 3% acetic acid) or Alcian blue/periodic acid-Schiff^52^,^53^ and acridine orange^25^. DNA counterstaining also was performed with Hoechst in deionized water. Uvitex-2B and calcofluor white are biological dyes that detect chitin^54^,^55^. Chitinase (*Streptomyces,* C6137, Sigma-Aldrich) treatment was performed for 1 h at 37 °C at 1 mg ml^−1^. Conditions for IHC and immunofluorescent antibody staining to VCBP and the human IgG1 Fc-CBD-C reagent utilized previously described methods^5^,^6^. Briefly, dewaxed paraffin tissue sections were incubated with 0.1% Triton/1 × PBS for 5 min and blocked in non-animal block buffer (Vector Laboratories, Burlingame, CA) for 1 h. Primary antibodies at appropriate dilutions were incubated overnight at 4 °C, and secondary antibodies were hybridized at room temperature for 2 h or overnight at 4 °C. Throughout, secondary antibodies were DyLight 488-conjugated (goat anti-human IgG1 for Fc-CBD-C detection; Invitrogen) and Alexa Fluor 594-conjugated (goat anti-rabbit IgG for VCBP-C detection; Abcam) and visualized with fluorescence microscopy.

### 16S fluorescent *in situ* hybridization

Detection of bacteria on biological sections was performed as described previously^11^,^56^. Briefly, tissue sections were permeabilized in 0.1% Triton/PBS for 5 min and treated with 0.1 M glycine for 10 min at room temperature. Prehybridization was at 37 °C for 1 h using a nonspecific blocking oligonucleotide. Hybridization of the sections using 50 ng of Cy3-conjugated 16S rRNA-specific universal oligonucleotide, EU338, was carried out overnight at 37–45 °C in hybridization buffer, 0.9 M NaCl, 20 mM Tris-HCl (pH 7.6), 0.01% SDS and formamide at 10–30%. A control sense oligonucleotide (non-EU338) was hybridized on corresponding sections.

### Production of hydrolysed chitin

Hydrolysed chitin was produced with minor modification of a previously described method^57^. Briefly, 20 g of chitin (crab shells, practical grade C7170; Sigma) was mixed with 200 ml of 12 N HCl and stirred for ∼15 h at 4 °C. The mostly dissolved suspension was adjusted to pH 8.4 using sodium carbonate, brought to a final volume of 1 l and autoclaved. Dilutions of the resulting buffered suspension were utilized in bacterial cultures and biofilm assays. VCBP-C binds to chitin prepared in this manner, which has been immobilized on columns^5^.

### Isolation and culturing of native gut bacteria

Fecal material was cleared by starving animals for 72 h, with repeated changes of 0.2-μm filtered seawater. Gut compartments (stomach, midgut and hindgut) were dissected, aseptically, from *Ciona* adults. A Dounce homogenizer was used to disrupt tissue and liberate or release bacteria from the mucosal surface of the gut compartments. Large tissue debris was eliminated by processing through a 40-μm filter at 500*g* for 30 s. Bacteria were isolated by pelleting at 1,500*g* for 10 min, washed once and resuspended in 0.2-μm filtered artificial seawater. Approximately 10–50 μl of the suspension was plated onto various media plates. Clonal growth was established and maintained by replica-plating and/or streaking. Bacterial classification is based on sequencing of 16S PCR products.

### *In vitro* biofilm assays

Bacterial isolates from fresh replica plates were grown in liquid culture at the appropriate temperature with continuous shaking or under stationary conditions; growth rates were determined by OD_600_. Cultures were diluted to a final concentration of 10^6^ cells per ml and plated (1 ml) onto 3-cm uncoated plastic Petri dishes in triplicate for stationary growth at 18 °C. Biofilms were developed for 2–5 days without agitation; excess liquid and planktonic (unbound) bacteria were removed by decanting or wicking with high-absorbance blotting pads cut into 0.5-cm strips, depending on the bacterial species and specific properties of the biofilm. The plates and adherent biofilms were dried in a biological safety cabinet for ∼2 h at room temperature, rinsed in water to remove loose material and stained in 0.1% crystal violet for 10 min (ref. 21). Biofilm production was estimated semiquantitatively by dissolving the stained material in ethanol or acetic acid using the same volume as the original culture and determining OD_550_. Biofilm staining levels of experimental groups were compared with those of parallel control groups using Dunnett's Multiple Comparison Test in InStat (GraphPad Software Inc., La Jolla, CA, USA). Statistically significant differences were presumed for *P* values<0.05. The observation that SIgA can enhance the formation of biofilms in a typical, non-pathogenic, version of *E. coli* (MG1655; ATCC 700926)^58^ was explored. *E. coli* was grown in stationary cultures in the presence of 0.5–2 mg ml^−1^ of SIgA (Thermo Fisher; 50-489-911); biofilms were induced most strongly at 2 mg ml^−1^ as previously shown^58^. *E. coli* was also cultured in the presence of 2 mg ml^−1^ recombinant VCBP-C. To visualize VCBP-C bound to established biofilms, bacterial cultures derived from the *Ciona* gut were grown in the presence of VCBP-C for 5 days and incubated with additional VCBP-C for 2 h before fixation. After the supernatant was removed, the biofilms were washed two times and fixed in paraformaldehyde and then washed an additional two times. Biofilms were stained with rabbit anti-VCBP-C and detected by goat anti-rabbit Alexa Fluor 594 (red). SIgA was detected with Alexa Fluor 488-conjugated AffiniPure F(ab′)2 Fragment Goat Anti-Human Serum IgA, α-Chain Specific (Jackson ImmunoResearch). Control biofilms were established without exposure to VCBP-C or SIgA and then stained with primary and secondary antibodies as before. Biofilm visualization was achieved with the air–liquid interface (ALI) assay^22^, modified to include visualization using a Leica stereoscope (M205 FA) and advanced optics, allowing up to × 320 magnification.

### RNA *in situ* hybridization

Whole-mount RNA *in situ* hybridization was performed on appropriately staged juveniles^5^,^6^. The expression of a putative chitin synthase gene (XM_004227126) from gut samples of unchallenged animals was verified using RT–PCR. A fragment of the chitin synthase transcript was amplified by PCR using S-5′-TTCGTCACTCAAGGCTGTTG-3′ and AS-5′-CGACACACACAATCCCTGTC-3′ oligonucleotides, cloned into TOPO vectors (Invitrogen) and riboprobes were generated with T3 and T7 polymerases according to the manufacturer's protocols. Endogenous alkaline phosphatase signal was quenched from gut tissues with an overnight incubation at 4 °C using 24 mg ml^−1^ of levamisole before developing with NBT/BCIP substrates (Roche Diagnostics, Indianapolis, IN).

## Additional information

**How to cite this article**: Dishaw, L. J. *et al.* Gut immunity in a protochordate involves a secreted immunoglobulin-type mediator binding host chitin and bacteria. *Nat. Commun.* 7:10617 doi: 10.1038/ncomms10617 (2016).

## Supplementary Material

Supplementary Information Supplementary Figures 1-7
