# Supplementary material for: Gut immunity in a protochordate involves a secreted immunoglobulin-type mediator binding host chitin and bacteria
Source: Nat Commun. 2016 Feb 15;7:10617. doi: 10.1038/ncomms10617 (PMC4757023; doi:10.1038/ncomms10617)
Supplement: Supplementary Information — Supplementary Figures 1-7 [file ncomms10617-s1.pdf]

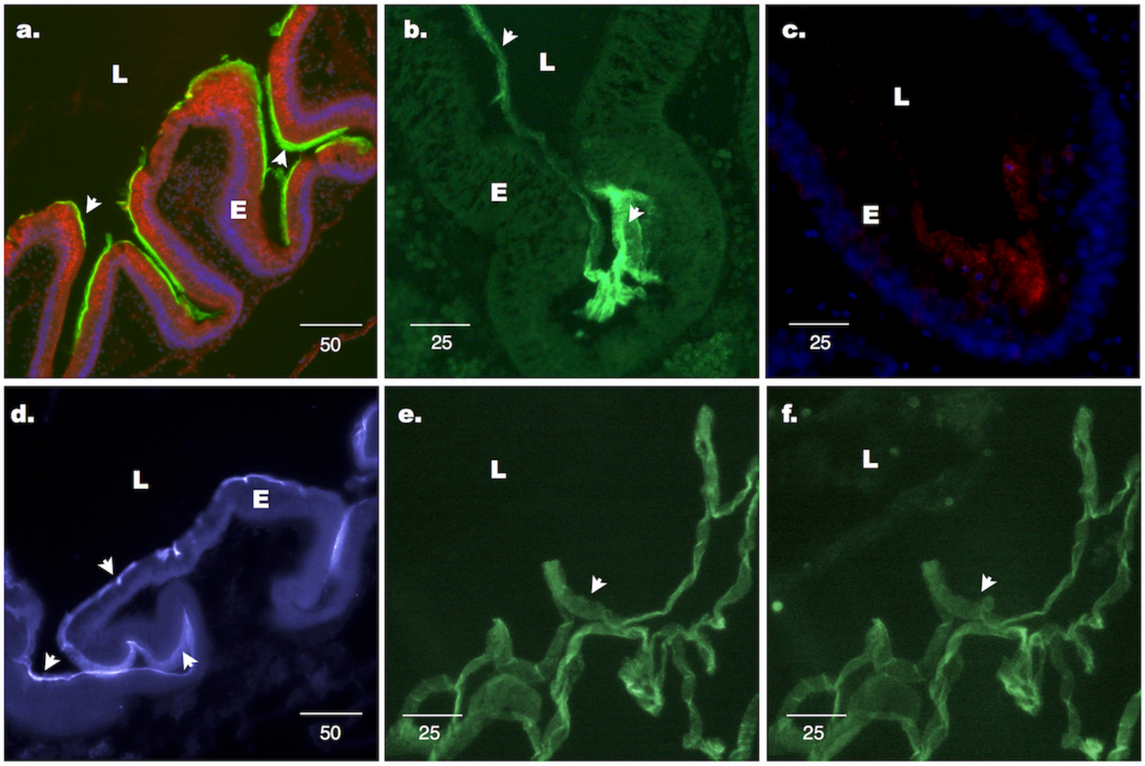

Supplementary Figure 1. Chitin-rich mucus on the surface of stomach epithelium. **(a)** A glycocalyx-like thin layer of chitin-rich mucus lines the stomach epithelial folds as revealed by Fc-CBD staining, detected by DyLight^{TM} 488 (green). Chitin-rich mucus collects at the base of the stomach epithelial folds or grooves **(b)** where VCBP-C is expressed most intensely (**c,** red), an effect noted previously (Liberti et al. 2014). This layer of mucus also stains with Calcofluor-white **(d)**, a biological stain for chitin. Longer chitin-rich fibers are noted exiting the stomach and into the midgut. All chitin-rich mucus can be detected with the Fc-CBD probe (**a, b, e**) and is mirrored in chitin staining with a commercial CBD probe (New England BioLabs; example in **f.**). Scale bars (micrometers) are indicated. E, epithelium; L, lumen.

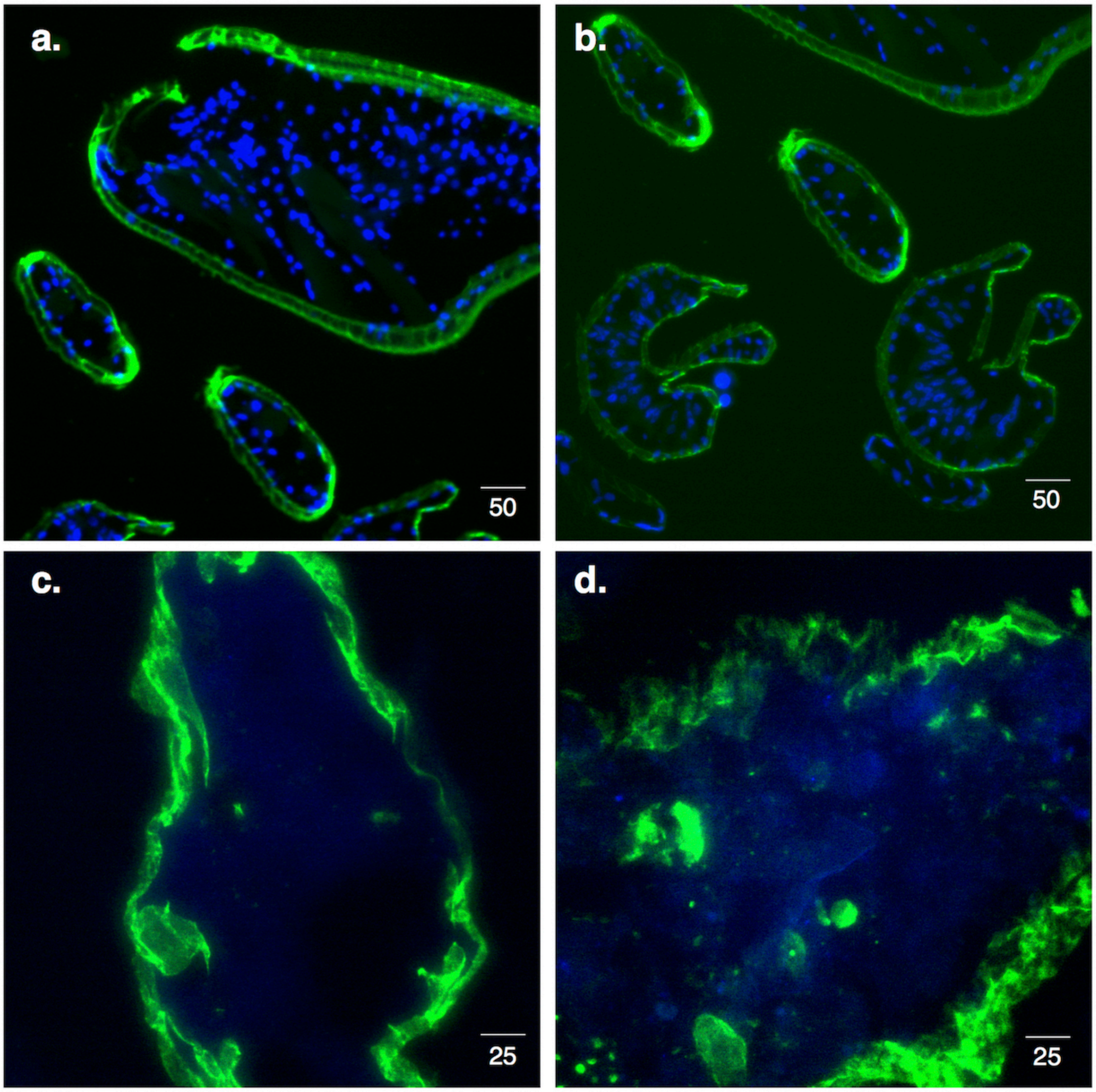

Supplementary Figure 2. Chitin-rich glycocalyx encases fecal pellets throughout the *Ciona* gut. In addition to lining the epithelial surfaces, a chitin-rich glycocalyx structure (green) surrounds food particles **(a,b)** and dense fecal pellets **(c,d).** Scale bars (micrometers) are indicated.

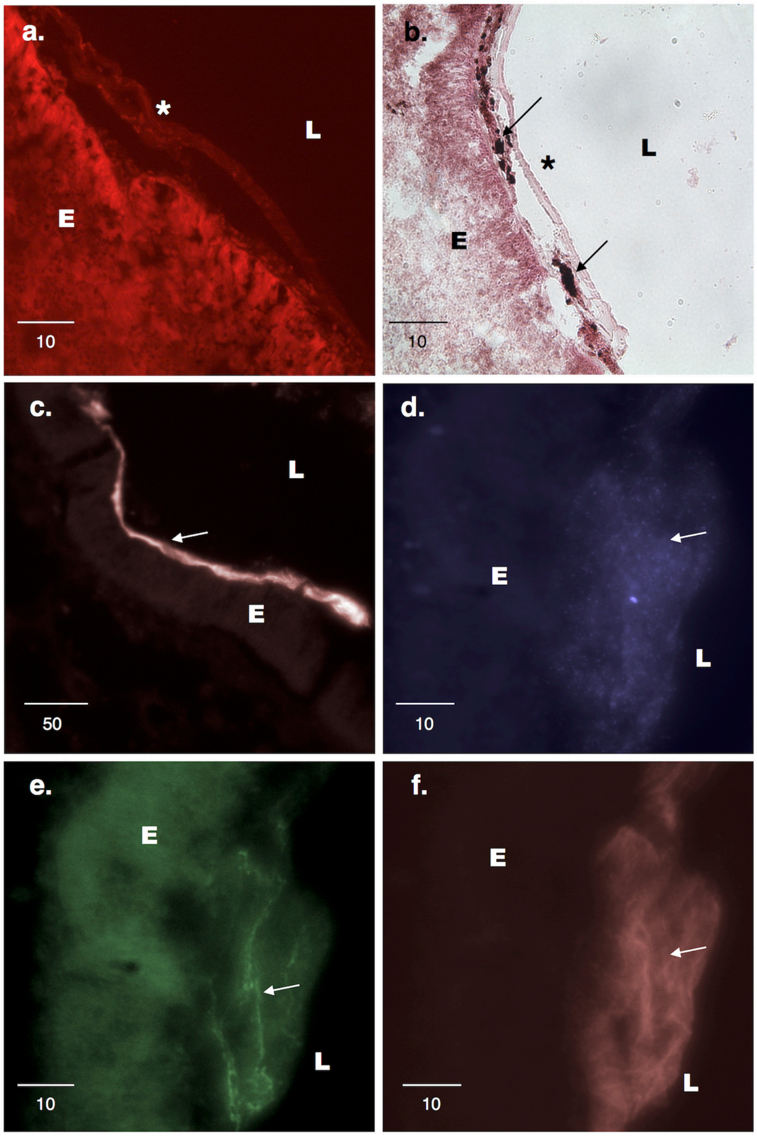

Supplementary Figure 3. VCBP-C co-localizes to glycocalyx-like layers and microbe-rich mucus in intestinal compartments. (a) In the gut, a thin rigid glycocalyx-like structure (∗) is detected by staining with acridine orange adjacent to the epithelium, which often reveals small microbe-sized structures. **(b)** VCBP-C, visualized by anti-VCBP-C detected with horseradish peroxidase-coupled secondary antibodies reacted with DAB (3,3'-diaminobenzidine) substrates (Vector Labs), often is detected under the glycocalyx. The signal from localized VCBP-C can be very prominent throughout the intestinal compartments in the flat dense **(c)** and less dense **(d-f)** mucus of the distal gut. **(d)** Microbe-sized objects were detected by Hoechst staining**. (e)** Chitin and **(f)** VCBP-C were visualized by immunofluorescent staining as noted before. Arrows emphasize stained mucus. Nuclei of intestinal epithelium are not seen in this plane of sectioning. Scale bars (micrometers) are indicated. E, epithelium; L, lumen; **(a-b)** frozen sections; **(c-f)** paraffin embedded sections.

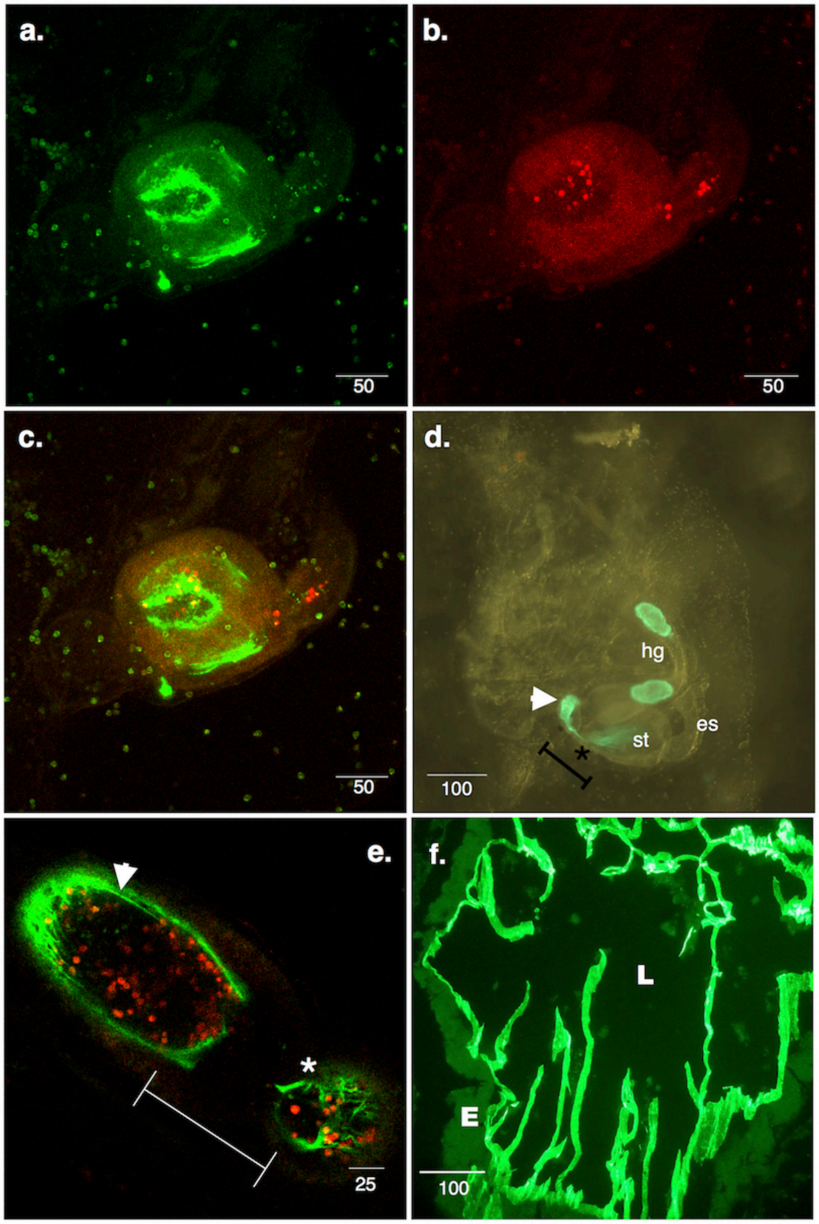

Supplementary Figure 4. Chitin rich mucus fills the stomach lumen and wraps fecal pellets. In more mature juveniles, chitin-rich mucus is seen to fill the stomach and leaves a narrow, empty space for food particles **(a).** A prominent signal is noted on the inner mucus surface and on the ventral side (arrows) of the stomach (as seen in **Fig. 5e**). This stomach mucus also is positive for VCBP-C **(b).** Merged signal is shown in **(c)**. Fecal material first becomes encased in the stomach lumen as they exit (***** in **d,e**). The formation of chitin-wrapped fecal pellets exiting the stomach is shown **(e).** A corresponding area of the adult gut sectioned and stained reveals long strands of chitin-rich mucus extending into the lumen **(f).** Confocal immunofluorescent microscopy is shown in **(a-c)** and **(e)** imaged with whole mount juveniles. VCBP-C (Alexa Fluor® 594, red) and chitin (DyLight^{TM} 488, green) are co-localized (yellow). Scale bars (micrometers) are indicated.

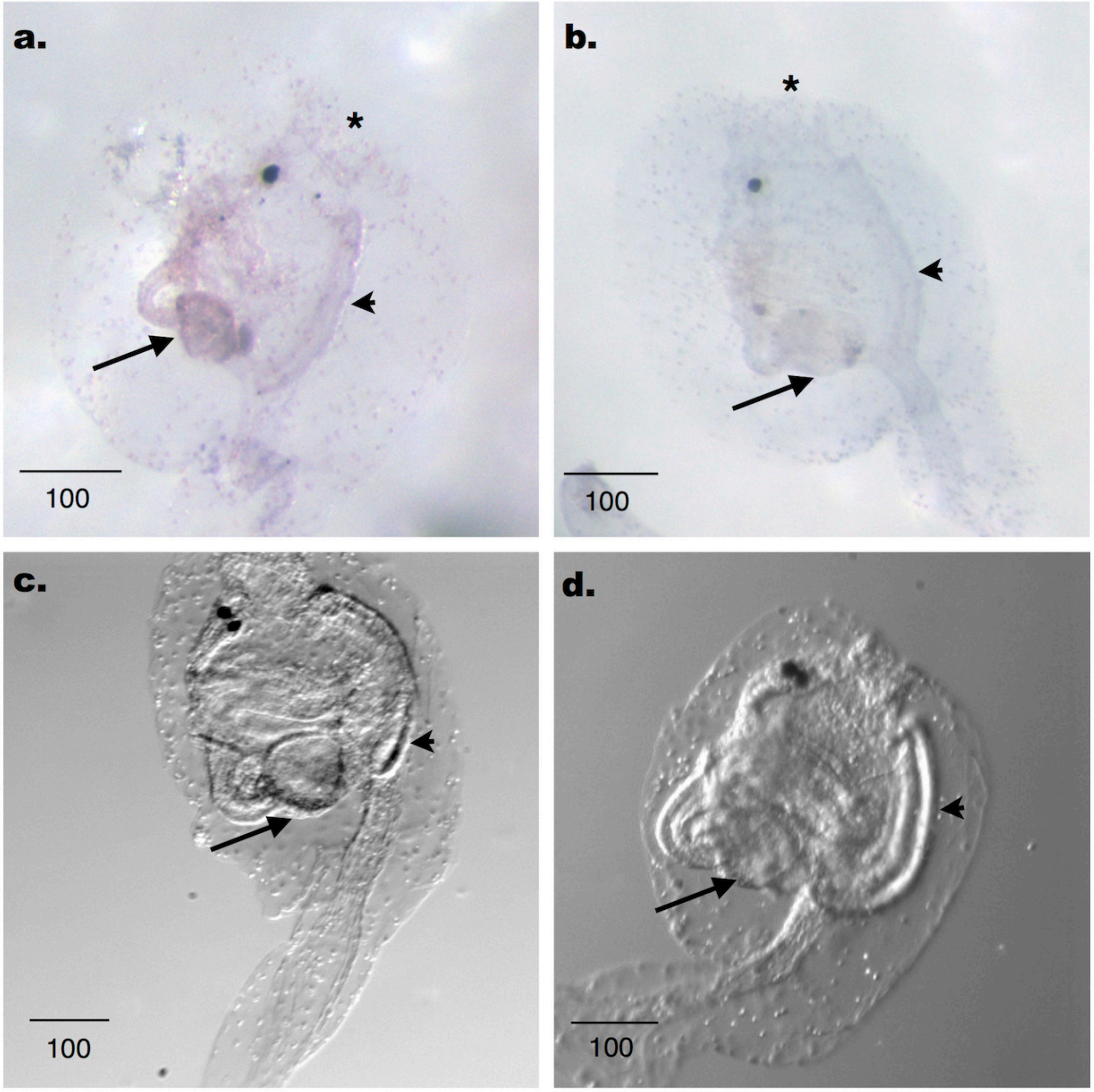

Supplementary Figure 5. Expression of chitin synthase in the stomach compartment of unfed, young juveniles maintained under germ-free conditions is detected by RNA *in situ* hybridization. (a) Chitin synthase can be detected in the stomach (arrows) of stage 5/7 juveniles that were maintained in germ-free conditions; **(b)** sense control. **(c)** Phase contrast microscopy reveals a predominant signal on the ventral surface of the developing gut, consistent with the signal noted in older animals **(Fig. 5e** and **Supplementary Fig. 4a,c)**; **(d)** sense control. The endostyle is indicated by arrowheads (for orientation purposes only). (*) main syphon; scale bars (micrometers) are indicated.

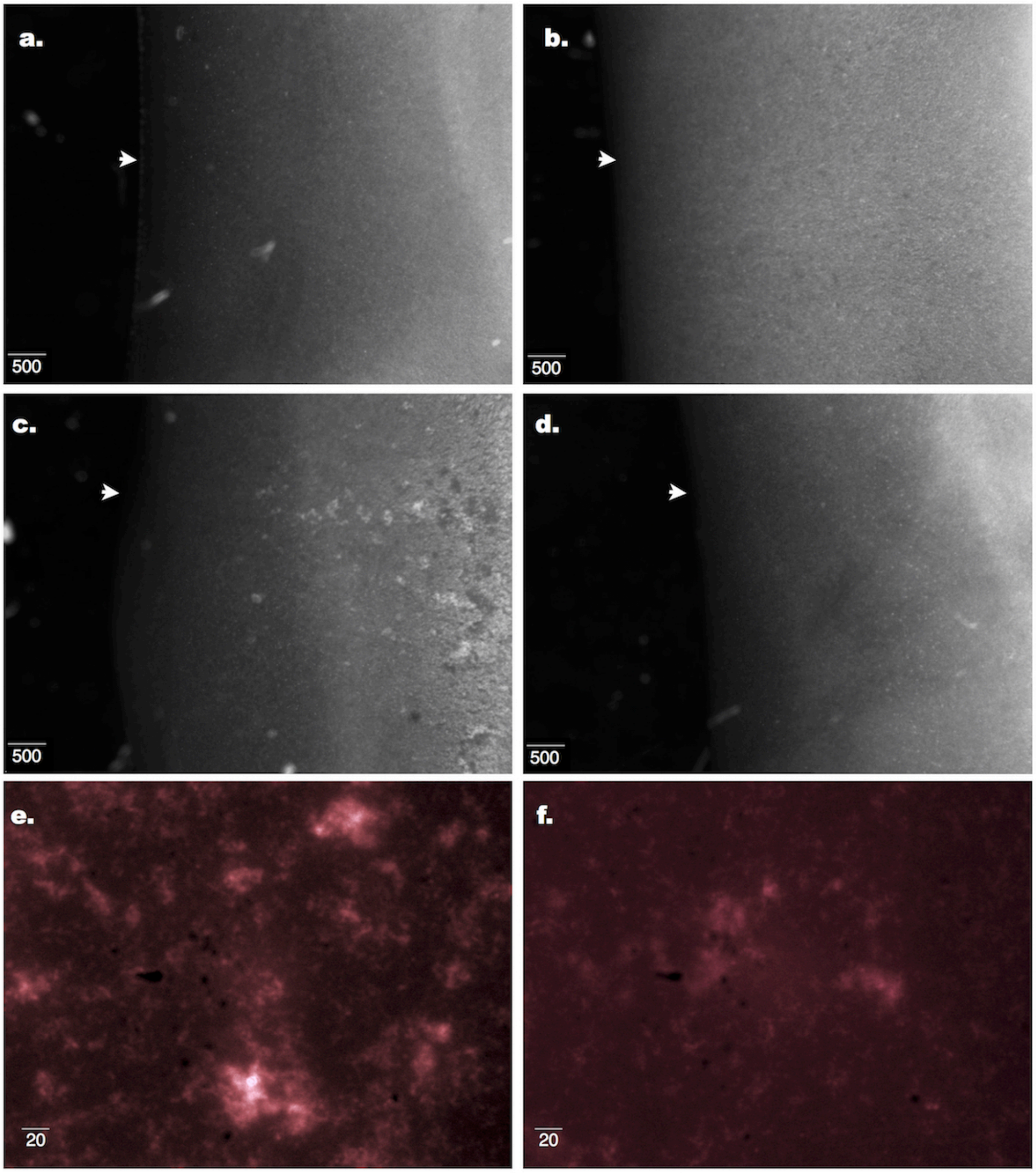

Supplementary Figure 6. Surface-coverage by developing biofilms. The Air-Liquid Interface (ALI) Assay is used to reveal surface coverage by developing biofilms. (**a)** Control (untreated) well from *Shewanella sp.* biofilm at 48hrs, compared to *Shewanella* co-cultured with full length VCBP-C **(b)**, co-cultured with full length VCBP-C and hydrolyzed chitin **(c)**, or co-cultured with the recombinant V1V2 protein **(d).** Enhanced biofilm surface coverage is indicated when co-cultured with full length protein **(b, c).** The effect in **(d)** is confirmed by immunofluorescence (**e**, co-culture with full length VCBP-C; **f**, co-culture with V1V2 protein). All cultures were plated in triplicate. Samples **(a-d)** were imaged from the same column of the 24 well dish at the same magnification, focal plane, and exposure settings. Arrowheads **(a-d)** identify the air-liquid interface of the biofilm. Scale bars (micrometers) are indicated.

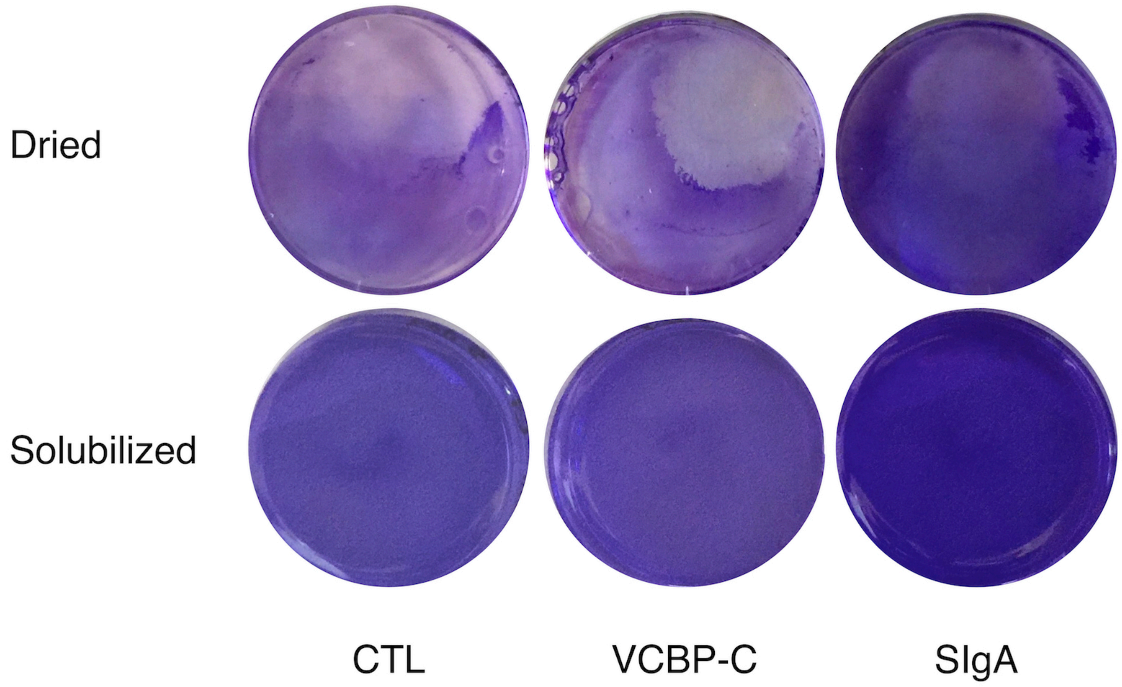

Supplementary Figure 7. Adherent biofilm formation in *Escherichia coli* is enhanced in the presence of SIgA but not VCBP-C. Crystal violet staining of dried biolims (top row) solubilized in acetic acid for quantification (see Fig. 6). Like SIgA, VCBP-C was incubated with *E. coli* at 2mg/ml for 48hrs in stationary cultures. Suspended culture is removed and the biofilm is washed two times and dried. Biofilms are stained in crystal violet. CTL, control (no protein added).
